# Supplementary material for: Nucleus accumbens controls wakefulness by a subpopulation of neurons expressing dopamine D1 receptors
Source: Nat Commun. 2018 Apr 20;9:1576. doi: 10.1038/s41467-018-03889-3 (PMC5910424; doi:10.1038/s41467-018-03889-3)
Supplement: Supplementary file 1 — Supplementary Information [file 41467_2018_3889_MOESM1_ESM.pdf]

Supplementary figures

Luo et al. Nucleus accumbens controls wakefulness by a subpopulation of neurons expressing dopamine D_1 receptors

Supplementary Figure 1

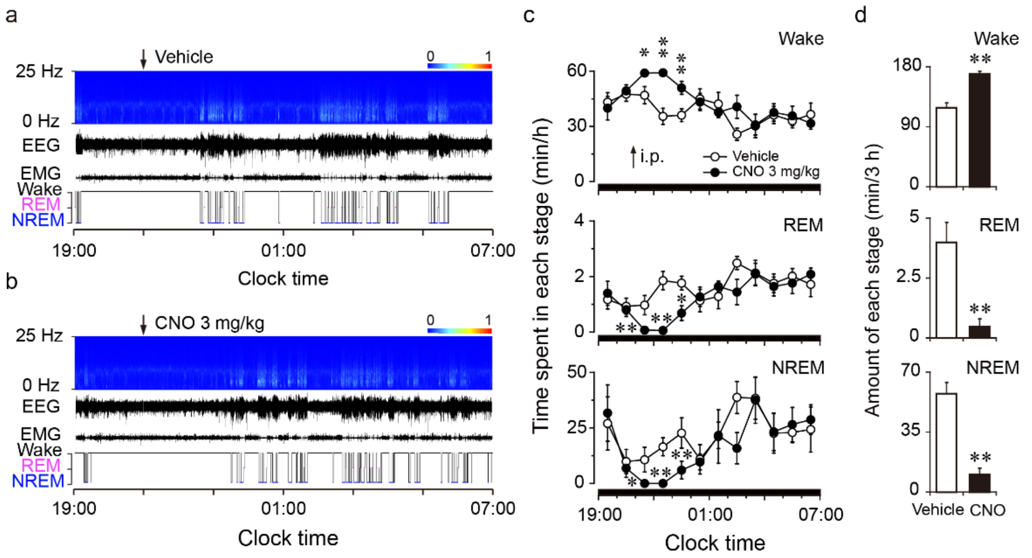

Supplementary Figure 1. Chemogenetic activation of NAc D_1R neurons increases wakefulness during the dark period. (**a, b**) Typical examples of an EEG power spectrum, EEG/EMG traces, and corresponding hypnograms illustrating effects of vehicle (**a**) or CNO (**b**) injection at 21:00 in a mouse expressing hM3Dq in NAc D_1R neurons. (**c**) Time course changes in wakefulness, REM sleep, and NREM sleep after administration of vehicle or CNO (3 mg/kg, i.p.) to D_1R-Cre mice expressing hM3Dq in NAc D_1R neurons. Data represent mean ± s.e.m. (n = 8). *P < 0.05, **P < 0.01, compared with vehicle control using repeated-measures ANOVA. (**d**) Total time spent in each stage for 3 h after administration of vehicle or CNO. CNO injection caused a significant increase in wakefulness for 3 h. Data represent mean ± s.e.m. (n = 8). **P < 0.01, compared with vehicle control by paired *t* test.

Supplementary Figure 2

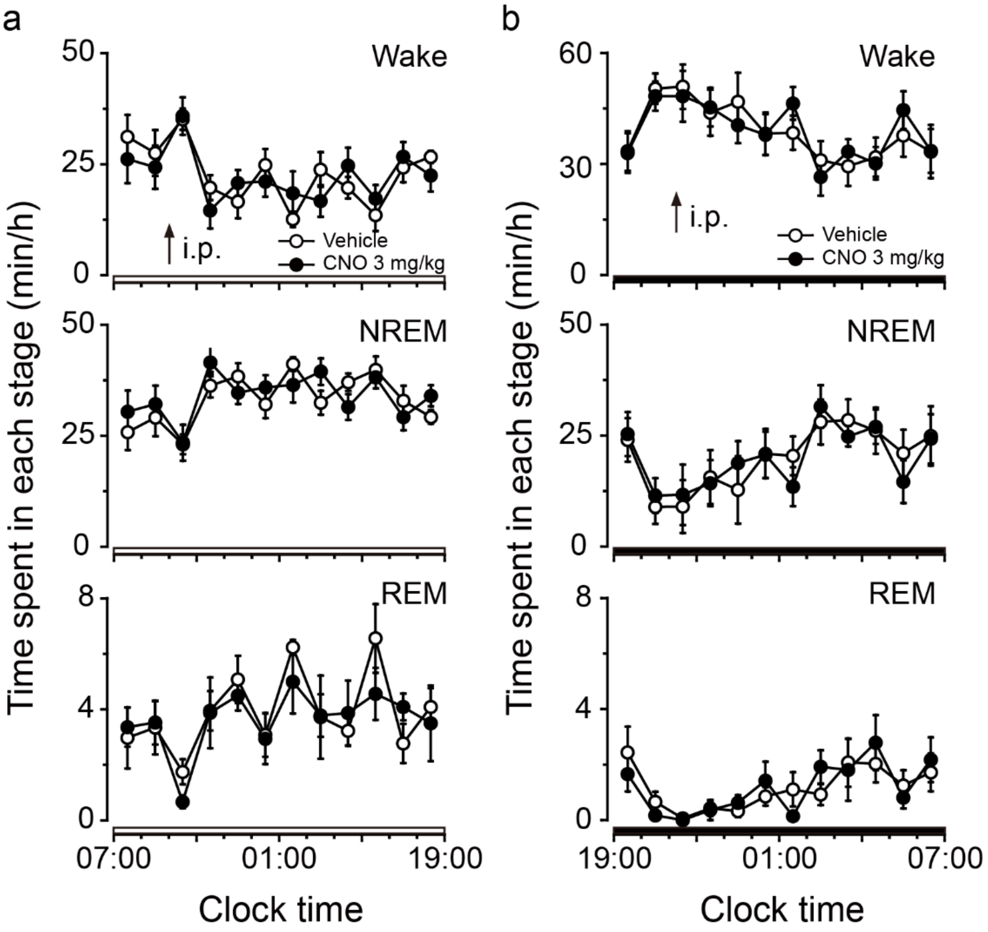

Supplementary Figure 2. Sleep–wake pattern following CNO administration in D$_1$R-Cre mice transduced with AAV-hSyn-DIO-mCherry in the NAc. (**a, b**) Time course changes in wakefulness, NREM sleep, and REM sleep after administration of vehicle or CNO (3 mg/kg, i.p.) to D$_1$R-Cre mice during the light (**a**) or dark period (**b**). Data represent mean ± s.e.m. (n = 6 or 7). [Light period: $F_{1, 10} = 0.60$, $P = 0.45$; dark period: $F_{1, 12} = 0.25$, $P = 0.63$]. No significant differences were found compared with the vehicle control using repeated-measures ANOVA.

Supplementary Figure 3

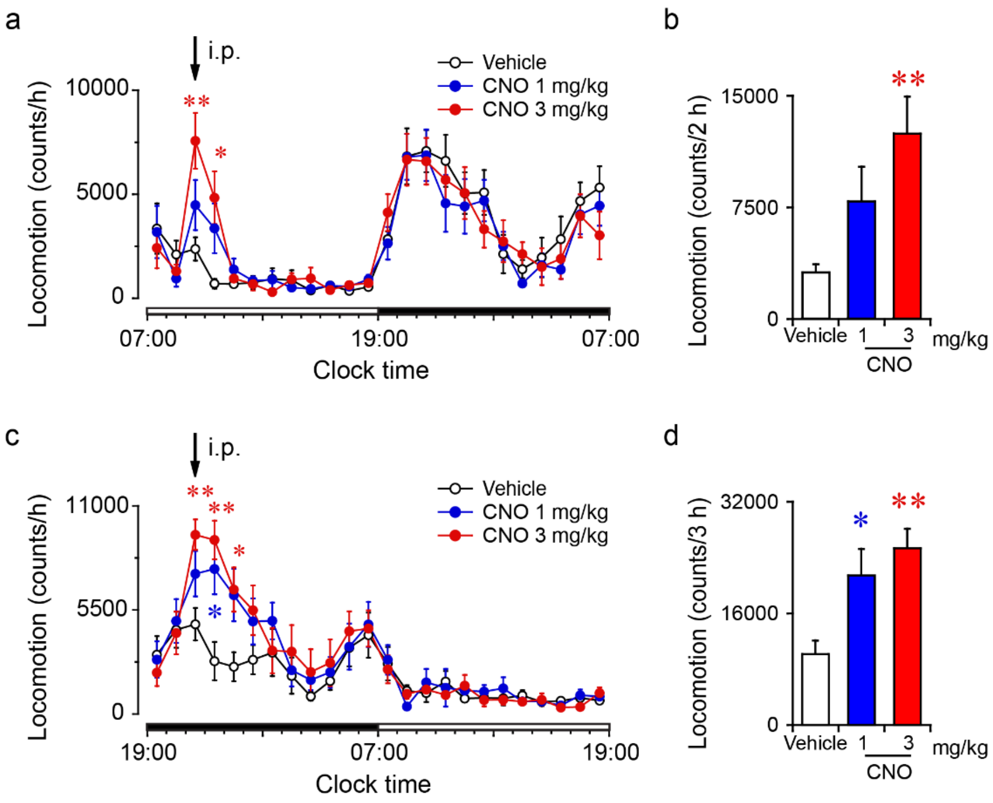

Supplementary Figure 3. Activation of D_1R neurons in the NAc increases locomotor activity during the light and dark periods. (**a, c**) Time course changes in locomotion after administration of vehicle or CNO (1 mg/kg, 3 mg/kg, i.p.) at 09:00 (**a**) or at 21:00 (**c**) to D_1R-Cre mice expressing hM3Dq in NAc D_1R neurons. Data represent mean ± s.e.m. (n = 8). *P < 0.05, **P < 0.01, compared with vehicle control using two-way repeated-measures ANOVA. (**b, d**) Total locomotion for 2 h or 3 h after administration of vehicle or CNO during the light (**b**) or dark (**d**) period. CNO injection caused a significant increase in wakefulness for 2 h during the light period or 3 h during the dark period [Light period: $F_{2, 21}$ = 5.47; dark period: P = 0.012; $F_{2, 21}$ = 7.18, P = 0.004]. Data represent mean ± s.e.m. (n = 8). *P < 0.05, **P < 0.01, relative to vehicle control as assessed by one-way ANOVA followed by by Turkey's post-hoc test.

Supplementary Figure 4

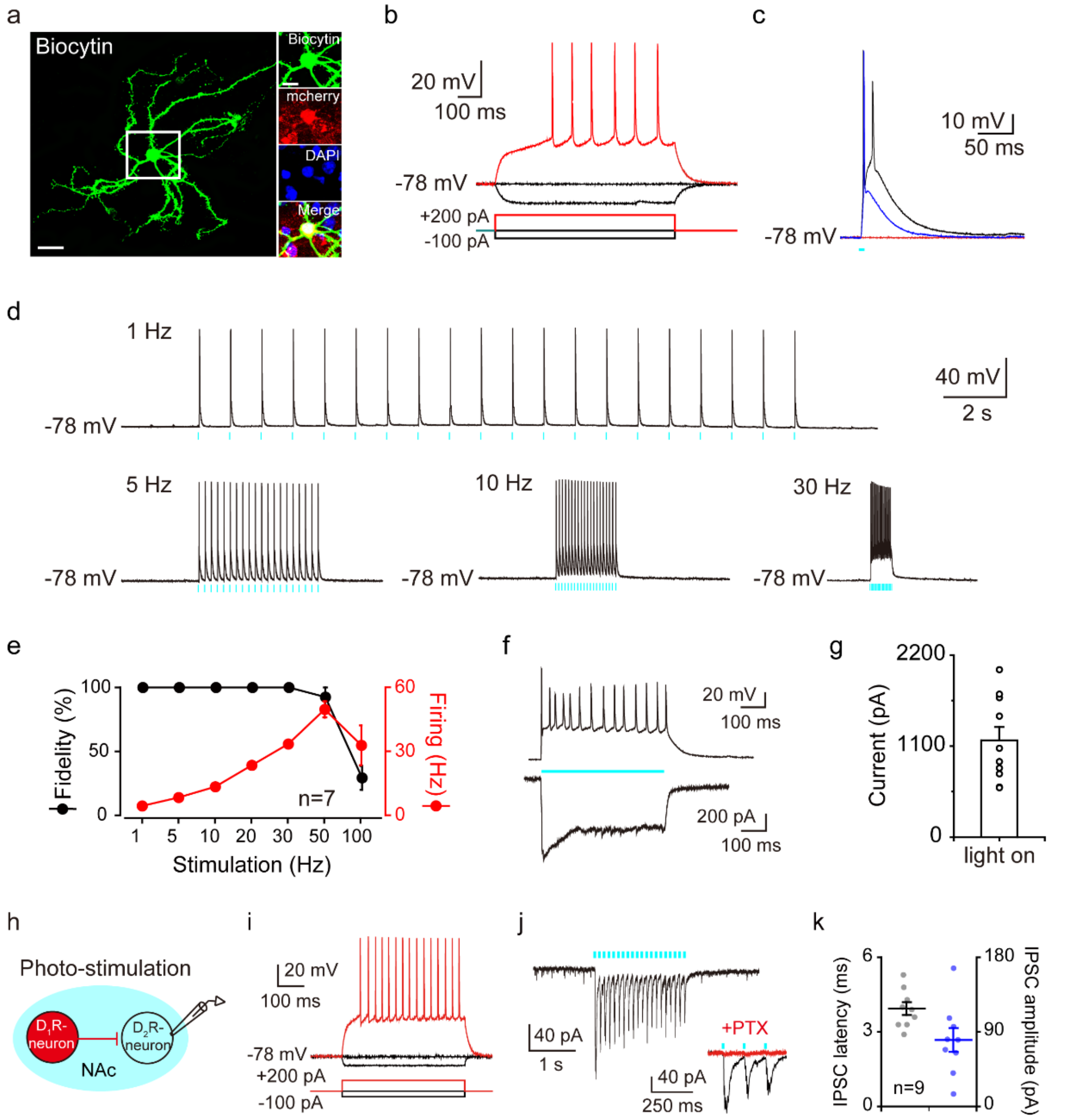

Supplementary Figure 4. Specific and efficient functional expression of ChR2 in NAc D_1R neurons. (**a**) Confocal image of a ChR2-mCherry (red) neuron filled with biocytin (green) in the NAc during whole-cell patch-clamp recording to visualize neuronal morphology. Scale bars: 20 μm. (**b**) Membrane potential changes induced by current injection into a ChR2-mCherry expressing D_1R neuron. RMP = -78 mV. Current steps: -100, 0, +200 pA. (**c**) Brief pulses (5-ms) of 473-nm light evoked single action potentials in ChR2-mCherry expressing neurons. Note that a pulse width > 15 ms typically resulted in spike doublets. (**d**) Group data showing fidelity of responses in ChR2-mCherry neurons following light pulses at frequencies up to 30 Hz. (**e**) Percentage change in spike fidelity (black, left axis) and ChR2-mediated entrainment of D_1R neuron firing (red, right axis) after 1–100 Hz photostimulation. Data represent mean ± SEM (n = 7). (**f**) ChR2-mCherry expressing neurons showed robust depolarization and spiking (top; RMP = -78 mV) following 500-ms blue light illumination in current-clamp mode. This depolarization coincided with inward current recorded under voltage-clamp (bottom; holding potential = -80 mV). (**g**) Quantification

of depolarization and inward currents in ChR2-expressing D_1R neurons following photostimulation (n = 10). (**h**) Schematic of the experimental design with an electrode indicating the cell type recorded. mCherry neuron expressing ChR2, red. Non-mCherry neuron, black. Blue oval indicates the area of photostimulation. (**i**) Membrane potential changes induced by current injection into a non-mCherry neuron (putative D_2R neuron). RMP = -78 mV. Current steps: -100, 0, +200 pA. (**j**) Representative traces of IPSCs evoked by laser stimulation recorded from a ChR2-negative neuron in the NAc; this current was PTX sensitive. PTX: picrotoxin, IPSC: inhibitory postsynaptic current. (**k**) Latency (black, left axis) and amplitude (blue, right axis) of $GABA_A$-mediated IPSCs in non-mCherry neurons (n = 9).

Supplementary Figure 5

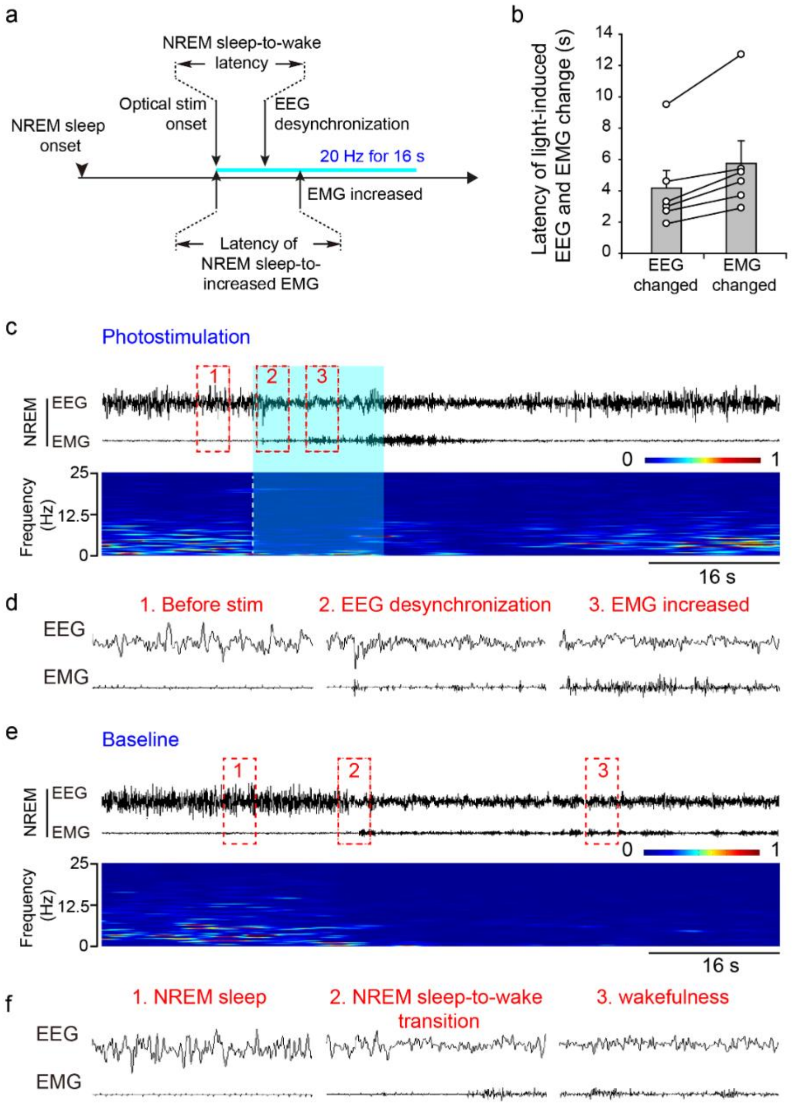

Supplementary Figure 5. Transient photostimulation of NAc D$_1$R neurons induces rapid EEG desynchronization followed by increased EMG activity. (**a**) Schematic of the experimental timeline. (**b**) Latencies to EEG or EMG change during NREM sleep following photostimulation (5-ms pulses at 20 Hz for 16 s) of NAc D$_1$R neurons in ChR2-mCherry-transduced mice. Column bar represents the mean ± s.e.m. NREM sleep-to-EEG desynchronization and NREM sleep-to-EMG increased latencies (4–9 trials per mouse, n = 6 mice). (**c, e**) Typical examples of EEG and EMG traces. EEG power spectrum illustrating the changing EEG power spectra and the raw EEG and EMG following photostimulation (**c**) or under basal conditions (**e**). The blue box indicates the timing of optical stimulation. Scale bars: 16 s. (**d, f**) Magnification of the boxes in c and e, respectively. Note the decrease in power of low frequency (< 4 Hz) oscillations that occurred a few seconds after the onset of optogenetic stimulation and were followed by increased EMG activity (**d**). Note the NREM-to-wake transition characterized by EEG desynchronization and increased EMG tone under baseline conditions (**f**). Stim: stimulation.

Supplementary Figure 6

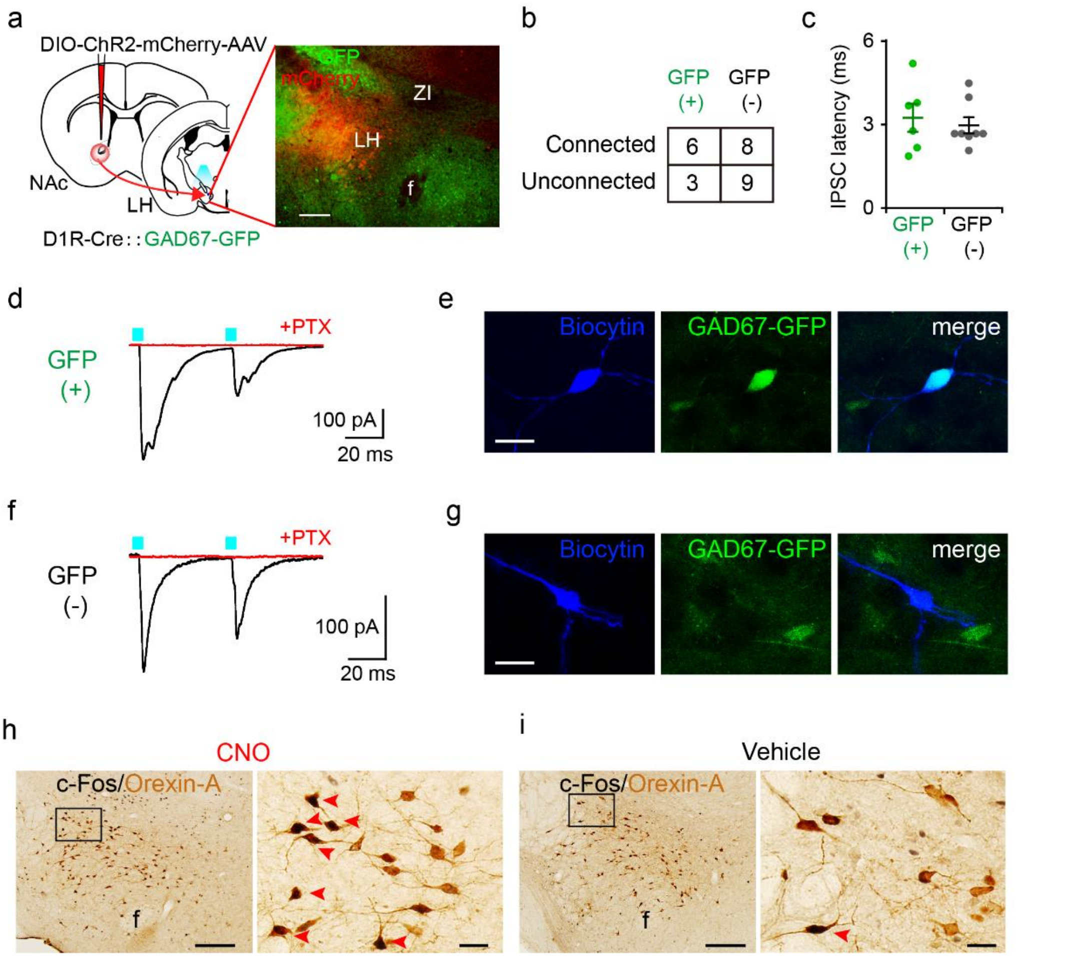

Supplementary Figure 6. NAc D_1R neurons innervate both GAD67-GFP positive and negative LH neurons. (**a**) Schematic showing injection of Cre-dependent ChR2 into the NAc of D_1R-Cre mice::GAD67-GFP mice. Terminals of the infected D_1R neurons were optogenetically activated and responses were recorded in LH neurons. The picture on the right shows the location of GAD67-GFP neurons (green) in the LH with ChR2-expressing fibers from NAc D_1R neurons (red). Scale bar: 200 µm. LH: lateral hypothalamus; ZI: zona incerta; f: fornix. PTX: picrotoxin. (**b**) Proportion of connected and unconnected neurons that were identified based on GFP expression (n = 9 GFP (+) neurons, n = 17 GFP (-) neurons, from 3 mice, Chi-square test; $\chi^2 = 0.875$, P = 0.429). (**c**) Latency of light-evoked IPSCs in LH GFP (+) neurons (left axis) and GFP (-) neurons (right axis). (**d-g**) Typical examples of a connected biocytin-labelled neuron that was GFP (+, **d, e**) and GFP (-, **f, g**) responsive to light stimulation. Scale bars: 20 µm. (**h, i**) Systematic injection of CNO in D_1R-Cre-hM3Dq mice induced robust c-Fos (black) expression in LH orexin (brown) neurons. Left, scale bar: 500 µm. Right, scale bar: 50 µm.

Supplementary Figure 7

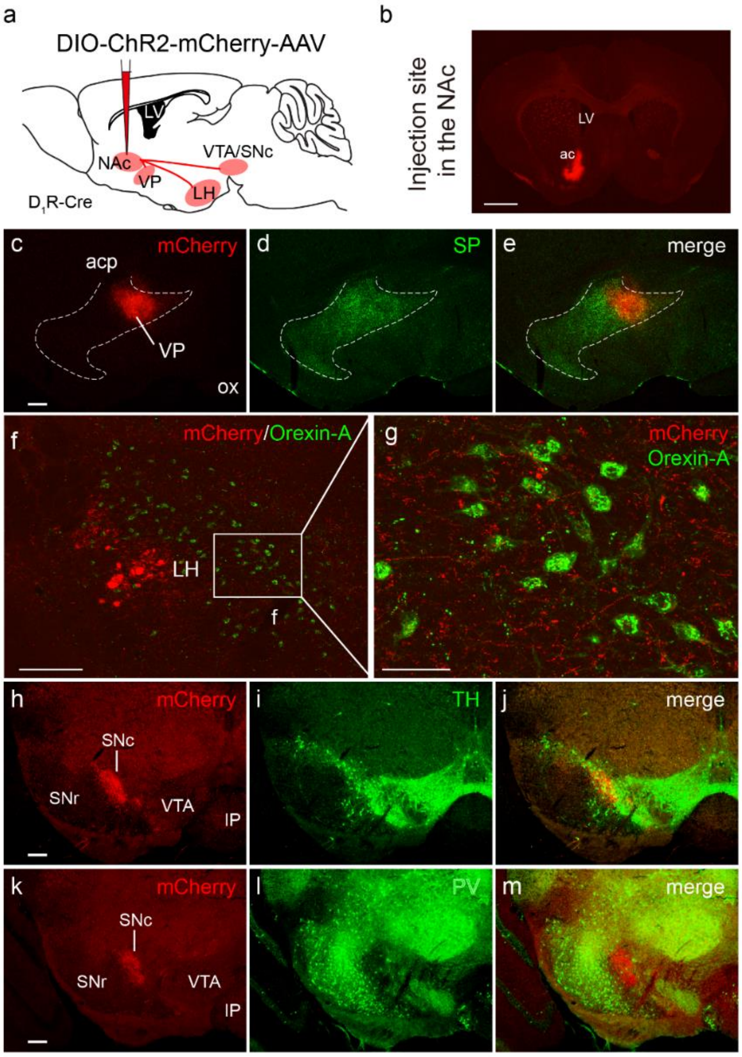

Supplementary Figure 7. Neural projection sites of NAc D_1R-expressing neurons. (**a**) Schema of ChR2-mCherry expressed in the NAc of D_1R-Cre mice. (**b**) Typical image showing the injection site of ChR2-mCherry in the unilateral NAc. Scale bar: 1 mm. (**c-e**) Prominent fiber terminals (red, **c**) are seen in the VP, was confirmed by co-staining for SP (defining borders of the VP, green, **d** and merged in **e**). Scale bar: 200 µm. (**f**) Low-magnification photomicrograph indicating the ChR2-mCherry axon terminals in a LH brain section immunolabeled against orexin-A (green). Scale bar: 200 µm. (**g**) High-magnification of boxed area in f. Scale bar: 50 µm. (**h-m**) Typical mCherry-positive fibers (red) in the midbrain (**h, k**), immunolabeling for TH identified dopaminergic neurons (green, **i**), and mCherry/TH was observed (**j**), immunolabeling for PV identified GABAergic neurons (green, **l**), and mCherry/PV was observed (**m**). Scale bars: 200 µm. LH: lateral hypothalamus; NAc: nucleus accumbens; SNc: substantial nigra pars compacta; VP: ventral pallidum; VTA: ventral tegmental area; ac: anterior commissure; acp: anterior commissure, posterior; LV: lateral ventricle; ox: optic chiasm; SP: substance P; f: fornix; IP: interpeduncular nucleus; SNr: substantial nigra reticular part; TH: tyrosine hydroxylase; PV: parvalbumin.

Supplementary Figure 8

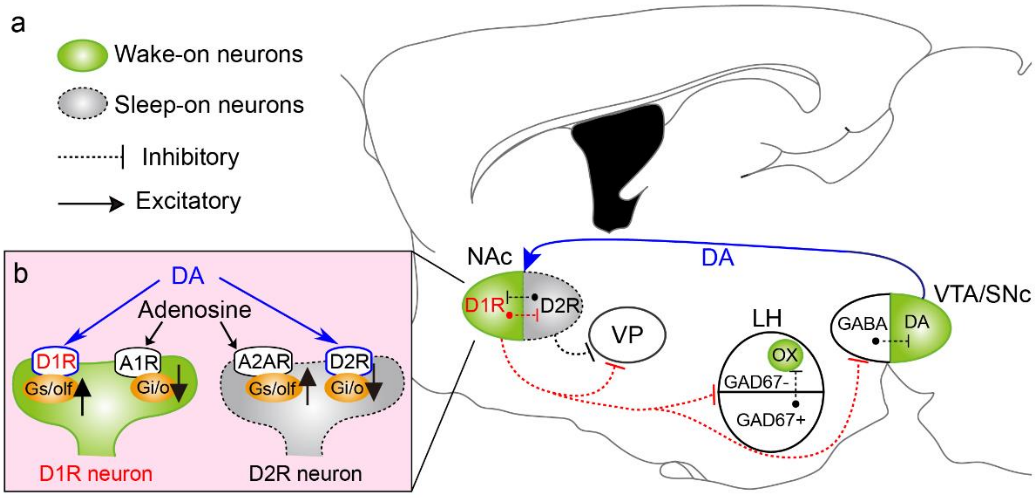

Supplementary Figure 8. Putative neural circuitry for NAc D_1R neurons controlling wakefulness. (**a**) Within the NAc, there exists collateral inhibition between D_1R neurons and D_2R neurons. NAc D_1R neurons may directly inhibit sleep-promoting D_2R neurons via local circuits. Both D_1R- and D_2R -neurons in the NAc project to the VP[1]. NAc $D_2R/A_{2A}R$ to VP pathway has been considered to be involved in sleep control[2], whereas NAc $D_2R/A_{2A}R$ to LH and VTA pathways are not important for sleep induction of NAc $D_2R/A_{2A}R$ neurons[2]. However, the role of NAc D_1R to VP pathway in sleep-wake regulation is needed to uncover. Activation of NAc D_1R neurons may excite LH orexin neurons and promotes wakefulness through disinhibition of orexin neurons. Inhibitory input from NAc D_1R neurons mainly target GABA neurons in the ventral midbrain (VTA/SNc), which may lead to disinhibition of wake-promoting DA neurons. VTA DA neurons also send dopamine projections to the NAc[3], thus the NAc and VTA are reciprocally connected, forming a NAc/GABA-midbrain/GABA-VTA/DA-NAc/GABA loop that controls wakefulness. (**b**) NAc D_1R neurons also express adenosine A_1 receptors, whereas D_2R neurons coexpress adenosine A_{2A} receptors. D_1Rs and $A_{2A}Rs$ are primarily coupled to Gs/olf proteins and stimulate the activity of adenylate cyclase, followed by PKA activation via cAMP accumulation. By contrast, D_2Rs and A_1Rs are associated with Gi/o proteins to inhibit cAMP production[4-6]. *In vitro and in vivo* electrophysiological studies indicate that D_1R activation modulates the intrinsic excitability of NAc neurons, and usually increases neuron activities, whereas D_2R activation often attenuates spike activities[7-9]. Thus, DA would differentially modulate D_1R- and D_2R- neuron activities through its action on excitatory D_1Rs or inhibitory D_2Rs, both of which are involved in wakefulness. On the other hand, adenosine promotes sleep through the activation of inhibitory A_1Rs and excitatory $A_{2A}Rs$. A_1R: adenosine A_1 receptor; $A_{2A}R$: adenosine A_{2A} receptor; D_1R: dopamine D_1 receptor; D_2R: dopamine D_2 receptor; DA: dopamine; GAD: glutamate decarboxylase; LH: lateral hypothalamus; NAc: nucleus accumbens; OX: orexin; SNc: substantial nigra pars compacta; VP: ventral pallidum; VTA: ventral tegmental area.

Supplementary Figure 9

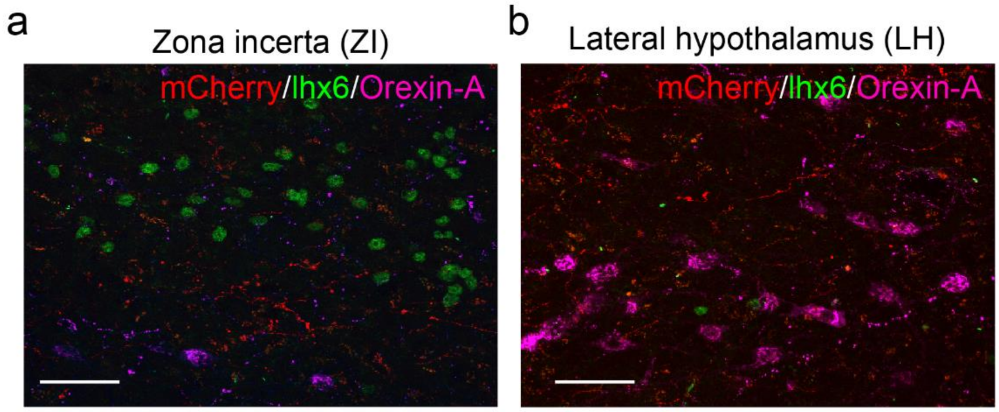

Supplementary Figure 9. NAc D_1R neurons send sparse fibers to ZI lhx6 (+) neurons. (**a, b**) Typical mCherry-positive fibers (red) in the ZI (**a**) and LH (**b**), immunolabeling for lhx6 identified a subpopulation of GABAergic neurons (green), and immunostaining for orexin-A identified orexin neurons (megenta). mCherry/lhx6/orexin-A was observed.

References:

1. Kupchik, Y.M., *et al.* Coding the direct/indirect pathways by D1 and D2 receptors is not valid for accumbens projections. *Nat. Neurosci.* **18,** 1230-1232 (2015).
2. Oishi, Y., *et al*. Slow-wave sleep is controlled by a subset of nucleus accumbens core neurons in mice. *Nat. Commun.* **8,** 734 (2017).
3. Eban-Rothschild, A., Rothschild, G., Giardino, W.J., Jones, J.R. & de Lecea, L. VTA dopaminergic neurons regulate ethologically relevant sleep-wake behaviors. *Nat. Neurosci.* **19,** 1356-1366 (2016).
4. Lazarus, M., Huang, Z.L., Lu, J., Urade, Y. & Chen, J.F. How do the basal ganglia regulate sleep-wake behavior? *Trends Neurosci.* **35,** 723-732 (2012).
5. Nagai, T., Yoshimoto, J., Kannon, T., Kuroda, K. & Kaibuchi, K. Phosphorylation signals in striatal medium spiny neurons. *Trends Pharmacol. Sci.* **37,** 858-871 (2016).
6. Lazarus, M., Chen, J., Urade, Y. & Huang, Z. Role of the basal ganglia in the control of sleep and wakefulness. *Curr. Opin. Neurobiol.* **23,** 780-785 (2013).
7. Perez, M.F., White, F.J. & Hu, X.T. Dopamine D(2) receptor modulation of K(+) channel activity regulates excitability of nucleus accumbens neurons at different membrane potentials. *J. Neurophysiol.* **96,** 2217-2228 (2006).
8. West, A.R. & Grace, A.A. Opposite influences of endogenous dopamine D1 and D2 receptor activation on activity states and electrophysiological properties of striatal neurons: Studies combining in vivo intracellular recordings and reverse microdialysis. *J. Neurosci.* **22,** 294-304 (2002).
9. Hopf, F.W., Cascini, M.G., Gordon, A.S., Diamond, I. & Bonci, A. Cooperative activation of dopamine D1 and D2 receptors increases spike firing of nucleus accumbens neurons via G-protein betagamma subunits. *J. Neurosci.* **23,** 5079-5087 (2003).
