# Supplementary material for: Nucleus accumbens controls wakefulness by a subpopulation of neurons expressing dopamine D1 receptors
Source: Nat Commun. 2018 Apr 20;9:1576. doi: 10.1038/s41467-018-03889-3 (PMC5910424; doi:10.1038/s41467-018-03889-3)
Supplement: Supplementary file 3 — Description of Additional Supplementary Files [file 41467_2018_3889_MOESM3_ESM.pdf]

Description of Additional Supplementary Files

File Name: Supplementary Movie 1
Description: Bilateral blue light stimulation of NAc D_1R neurons induced wakefulness from NREM sleep in a mouse expressed ChR2-mCherry in NAc D_1R neurons.

File Name: Supplementary Movie 2
Description: Bilateral blue light stimulation of NAc D_1R neurons did not induce wakefulness in a mouse transduced with mCherry in NAc D_1R neurons.

File Name: Supplementary Movie 3
Description: Unilateral blue light stimulation of left NAc induced wakefulness in a mouse expressed ChR2-mCherry in NAc D_1R neurons.

File Name: Supplementary Movie 4
Description: Bilateral blue light stimulation of terminals in the midbrain from NAc D_1R neurons induced wakefulness.

File Name: Supplementary Movie 5
Description: Bilateral yellow light stimulation of terminals in the midbrain from NAc D_1R neurons did not induce wakefulness.

File Name: Supplementary Movie 6
Description: Bilateral blue light stimulation of terminals in the lateral hypothalamus from NAc D_1R neurons induced wakefulness.
